# Exploring bioactive compounds from a symbiotic bacterial strain of Spongiobacter sp.

**DOI:** 10.6026/97320630019369

**Published:** 2023-04-30

**Authors:** Fehmida Bibi, Muhammad Imran Naseer, Esam Ibraheem Azhar

**Affiliations:** 1 Special Infectious Agents Unit-BSL3, King Fahd Medical Research Centre, King Abdulaziz University, Jeddah, 21589, Saudi Arabia; 2 Center of Excellence in Genomic Medicine Research (CEGMR), King Abdulaziz University, Jeddah 21589, Saudi Arabia; 3 Department of Medical Laboratory Sciences, Faculty of Applied Medical Sciences, King Abdulaziz University, Jeddah, 21589, Saudi Arabia

**Keywords:** Red Sea, *Pione vastifica*, 16S rRNA gene sequence, *Spongiobacter sp*, EA276, bioactive metabolites

## Abstract

Marine sponges are a host of different symbiotic groups of bacteria playing crucial roles in the protection and survival of marine sponges. Marine symbiotic bacteria from sponges are promising sources of bioactive chemicals and are increasingly being investigated. Therefore, the present study was undertaken to analyze total compounds from active symbiotic bacterial strain from sponge, *Pione vastifical*. Potential bacterial strain EA276 previously isolated from P. *vastifical* and was identified as *Spongiobacter* sp. Among 57 isolates, only 42% exhibited antagonistic activity. Four major classes of bacteria were reported previously where *γ-Proteobacteria*, was the dominant class. From these active antagonistic bacterial isolates, a potential bacterial strain *Spongiobacter* sp. EA276 was selected, and total metabolites were identified using GC and LC-MS analyses. Using LC-MS analysis bioactive compounds Dichlorphenamide, Amifloxacin and Carbenicillin are identified in both positive and negative mode. Plant growth hormones, Indole-3-acetic acid and Methyl jasmonate were identified using GC-MS analysis from culture extract of strain *Spongiobacter* sp. EA276. Our results highlighted the significance of marine flora inhabiting sponges from the Red Sea as potential source of bioactive compounds and plant growth hormones of biological and agricultural significance.

## Background:

Emerging infectious diseases become a serious health risk to human population and increasing in global prevalence of multidrug-resistant bacteria. Discovery of antimicrobial compounds is needed to treat public health-threating infectious diseases. Therefore, there is a need to combat these human health threats by discovering new antimicrobial compounds. The marine environment covering 70% of the earth's surface is a home of diverse bioactive molecules found in fauna and flora of marine environment [1]. Symbiotic microorganisms are important to protect the host against different pathogens by bioactive peptides. The oceans present a complex and unique environment where life development under hostile conditions of temperature, pressure and salinity allowed the synthesis of bioactive molecules from different marine organisms [2]. Symbiont microorganisms, especially bacteria, are producers of bioactive compounds that are diverse and unique in structure and function. These bioactive compounds have shown biotechnological properties such as anti-inflammatory, antimicrobial, antiviral, antitumor, antioxidant, anti-fouling, antiprotozoal and many other properties and other functions pharmaceutical, and medical significance [3]. Sponges are simple invertebrates with soft and sessile bodies lacking defensive features such as spines, spikes [4]. Sponges are a host and shelter for diverse mutualistic microbial communities. These microbes defend their host by producing different bioactive compounds and play an important role in survival and protection of host against predators [5]. Symbiotic bacteria play an essential role in chemical defense of host against predators by producing bioactive metabolites. This phenomenon of symbiotic microbes helps the host in their survival in the ecosystem. These secondary metabolites are part of the chemical defense system of sponges that is believed to be an essential ecological function for the protection of the host. Therefore, sponge-associated microbes gain attention and attracted many researchers to study these microbes and unravel bioactive molecules from them [6]. It is necessary to culture and identify these potential symbiotic microbial communities from sponges to understand their phylogeny and function as symbionts. Using culturomics, 11 bacterial phyla were retrieved from marine sponges where four phyla i.e., *Proteobacteria*, *Actinobacteria*, *Firmicutes*, and *Bacteroidetes* were dominated [7]. Our previous study using culture dependent techniques identified diverse communities of bacteria from marine sponge *P. vastifica* [8]. High percentage of bacteria showed antifungal and antibacterial activity. Therefore, it is of interest to study selective potential strain of bacteria i.e., *Spongiobacter* sp. EA276 from sponge *P. vastifica* isolated in our previous study. We also aim to identify metabolites of these potential strains using GC and LC-MS analysis.

##  Materials and Methods:

## Sample collection, Isolation, antagonistic activity and identification:

These sponge samples were collected from Obhur region in Jeddah, Red Sea at the depth of 40m. Identification, isolation techniques and culture conditions were mentioned previously [8]. Potential strains were tested against plant and human pathogenic bacterial strains *Escherichia coli* ATCC 8739, (Methicillin-resistant *Staphylococcus aureus* (MRSA) ATCC 43300, *Enterococcus faecalis* ATCC 29212), *Pseudomonas aeruginosa*f ATCC 27853, and oomycetes pathogens i.e., *Phytophthora capsici* and *Pythium ultimum*. Further selective strains were identified by performing 16S rDNA gene analysis [8]. EzTaxon server (https://www.ezbiocloud.net) was used for blast search and identification of strains [9]. For phylogenetic analyses, CLUSTAL_X version 1.83 [10] was used for aalignments of 16S rRNA gene sequences of active strains and closely related type strains. BioEdit software version 4.7.3 was used for editing of gaps between sequences [11]. Finally, MEGA6 Programme, was used where phylogenetic tree was generated using neighbor-joining method [12].

## Bacterial culture conditions optimization and identification of metabolites from crude extract:

Strains showing potential antimicrobial activity against tested bacterial and oomycetets pathogens and low 16S rRNA sequence similarity were selected for identification of their active metabolites. Strain EA276 from sponge *P. vastifica* was selected for identification of secondary metabolites from culture extract. Culturing conditions for selective strain were optimized by using different culturing media i.e., ½ R2A, Marine broth, and ½ TSB. At different incubation times (24hrs, 36hrs and 72hrs) optical density (OD) was tested. Antimicrobial activity of the culture from strains EA276 was checked after every 24hrs against oomycetes pathogens mentioned above. Temperature conditions (25-40°C) and pH conditions (6-12) were optimized. After defining optimized culture conditions, selective strains were grown for 36hrs and 5ml of bacterial culture was further processed first for 5mins at -70°C, then at 37°C. This process was repeated many times and was centrifuged at 12000-13000g (15mins). After centrifugation, 3ml of supernatant was mixed with acetonitrile (10ml) and vortexed (50sec) vigorously. Centrifugation is performed again at 13000g (15 mins) and 500µl of supernatant was further used for LC-MS analysis. Samples were analyzed as stated previously [13]. Raw data was further processed using Agilent Mass Hunter (version B.06.00) for further analysis. Metabolites from selective strains were identified using in-house database. Using Gas-chromatography mass spectrometry (GC-MS) metabolites were further analyzed using Shimadzu GCMS-QP2010 Ultra as described [13].

## Statistical analysis:

Different databases (SciFinder, ChemSpider, ChEMBL, PubChem, and National Institute of Standards and Technology (NIST) databases were used for identification of metabolites from selective bacterial strains.

## Results:

## Antimicrobial activity of selective bacterial strains:

Strain EA276 from sponge *P. vastifica* showed broad antimicrobial activity and was selected for further metabolites identification. Strain EA276 showed activity against oomycetes pathogens only and was negative for antibacterial activity (Table 1).

## Phylogenetic diversity of antagonistic bacteria:

**Figure 1:**
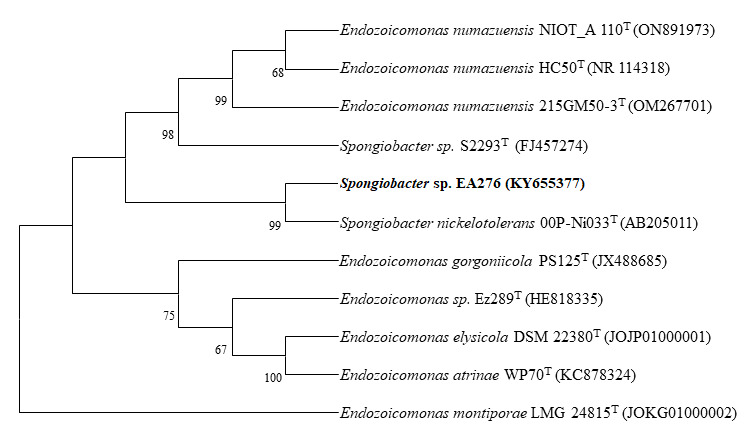
Phylogenetic distribution of bacteria closely related to *Spongiobacter* sp. EA276. The phylogenetic relationships were inferred from the 16S rRNA gene by using the neighbor-joining method from distances computed with the Jukes-Cantor algorithm. Bootstrap values (1,000 replicates) are shown next to the branches. GenBank accession numbers for each sequence are shown in parentheses. Bar, 0.01 accumulated changes per nucleotide.

Antagonistic bacterial strain *Spongiobacter* sp. EA276 was analyzed phylogenetically. The relationships of the *Spongiobacter* sp. EA276 was unveiled based on 16S rRNA gene sequences of the closely related strains. Neighbor Joining (NJ) phylogenetic tree for *Spongiobacter* sp. EA276 and related bacterial strains were constructed using 16S rRNA gene sequence data (Figure 1). *Spongiobacter* sp. EA276 showed close branching clad with species of genus *Spongiobacter* and genus Endozoicomonas species. Bootstrap or branch values were high for all species in phylogenetic tree.

## Identification of bacterial metabolites using LC-MS and GC-MS analyses:

**Figure 2:**
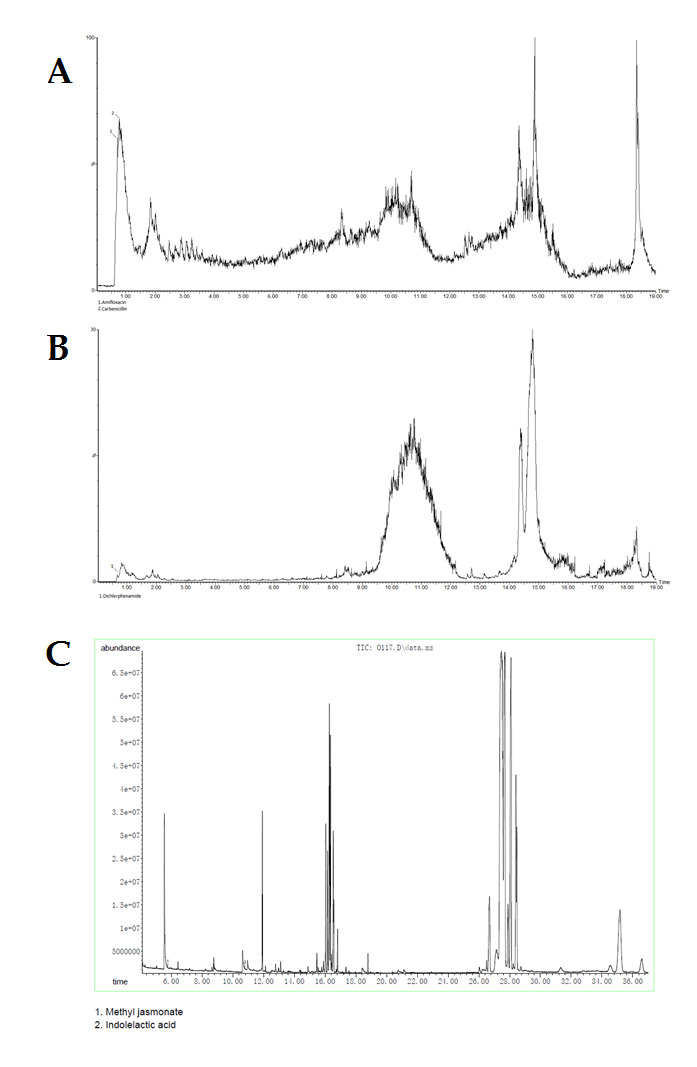
Bioactive secondary metabolites detected in culture extract of strain Spongiobacter sp. EA276. by LC/MS analysis (a) Positive mode and (b) negative mode and (c) by GC/MS analysis.

Selected bacterial strain EA276 was analysed for identification of secondary metabolites. For maximum yield of antimicrobial compound and its inhibitory activity bacterial culture conditions were optimized. From different tested media and conditions, it showed maximum inhibition against tested pathogens in modified ½ R2A broth at 28°C and pH 7.5. Identification of metabolites was done using both GC and LC-MS analyses. Different metabolites including some bioactive molecules were identified using both analyses from *Spongiobacter* sp. EA276 (Figure 2a-b). LC-MS analysis showed presence of four secondary bioactive compounds in both positive and negative mode (Figure 2a and b). These compounds include Dichlorphenamide, Amifloxacin and Carbenicillin. By using GC-MS analysis, peaks of some plant growth hormones such as Indole-3-acetic acid and Methyl jasmonate were detected from culture extract (Figure 2c).

## Discussion:

Sponges are sessile multicellular organisms harboring diverse bacterial symbionts [14]. Microbial symbionts are diverse, species-specific and play a pivotal role in persistence of sponges by playing functional roles such as production of secondary metabolites, nutrient cycling, photosynthesis and production of vitamins [15]. In the present study, antagonistic bacterial strain EA276 isolated from marine sponge *P. vastifica* was studied for identification of bioactive compounds. Our results revealed the presence of potential bioactive compounds and plant growth promoting hormones from strain studied. Sponges associated bacterial communities are essential for their host survival by producing secondary metabolites. These secondary metabolites are chemically diverse and exhibit different activities such as antimicrobial, anti-inflammatory, antiprotozoal, antiviral, anti-cancer and many other essential functions. These bioactive compounds belong to different groups of chemical compounds mainly, cyclic peptides, nucleosides, peroxides, alkaloids, bioactive terpenes, sterols and alkaloids fatty acids [16]. *P. vastifica* is a red boring sponge and is a species of demosponge found from the Red Sea to western Pacific Ocean. In our previous study, 24 antagonistic bacteria were observed from *P. vastifica* belonging to 3 different classes i.e., *γ-Proteobacteria*, *Firmicutes* and *Flavobacteria* [8]. In this study, *γ-Proteobacteria* was the dominant class of bacteria encompassing eight different genera including *Spongiobacter*. Both culture dependent and independent techniques have reported *Proteobacteria* as a dominant bacterial phylum from marine sponges. Members of the α- and *γ-Proteobacteria* are the most abundant bacteria producing antimicrobial compounds from marine sponges [17, 18]. We also studied an antagonistic bacterial strain *Spongiobacter* EA276 from phylum *γ-Proteobacteria* for identification of active metabolites. Our analysis confirmed the presence of active metabolites including antimicrobial compounds and plant growth hormones from culture extracts. The presence of these compounds from *Spongiobacter* sp. EA276 associated with *P. vastifica* confirmed and highlighted the ecologically important role of microbial symbiont. Antimicrobial compounds are considered to present a selective advantage and are produced for survival of producer strain in competition with other bacteria population. These metabolites prevent phagocytosis by predators and established a symbiotic interaction with their hosts [19]. *Spongiobacter* EA276 demonstrated its abilities that may be exploited in various biotechnological applications. Sponge associated bacteria, *Actinobacteria*, fungi and cyanobacteria were found to be the sources of antimicrobial and other bioactive metabolites in marine environment [16]. Previous studies using culture dependent and independent techniques showed *Spongiobacter* sp. as a dominant member of the sponge and coral-associated microbial community indicates that *Spongiobacter* play a significant role in the functioning system of associated host [20, 21]. Their role as potent producers of antimicrobial agents has also been recorded earlier [22]. Our studies confirmed their role as producer of bioactive compounds and identified their key role in sustaining sponge health. *Spongiobacter* was reported as a dominant group of bacteria from marine invertebrates and sponges where most of the isolates showed antimicrobial activity [22]. Using both GC and LC-MS analyses, Dichlorphenamide, Amifloxacin, Carbenicillin, Rescinnamine, Indole-3-acetic acid and Methyl jasmonate were identified.

## Conclusion:

To our knowledge, this is one of the first reports that identified potentially new compounds from this genus. No reports or data are yet available regarding the identification of compounds from sponge, *P. vastifica* or from its symbiotic bacterial population or from bacteria genus *Spongiobacter*. This potential strain of bacteria plays an essential role in the defense of the host sponge *P. vastifica* against different pathogens. It also highlighted the functional role of symbiotic bacteria in sponges as a promising source for the discovery of bioactive compounds. These results demonstrated that *Spongiobacter* EA276 associated with marine sponge P.vastifica could be used in future as a biocontrol agent against pathogens to control different diseases.

## Figures and Tables

**Table 1:** Taxonomic identification, antifungal and antibacterial activity of bacterial strain Spongiobacter sp. EA276 from sponges, *P. vastifica*

				**Antifungal activityc**		**Antibacterial acitivityd**			
Lab no	Accession Number	Similarity with closest type straina	% identityb	*P. capsici*	*P. ultimum*	*P.aeruginosa*	*S. aureus*	*E.coli*	*E.faecalis*
									
*P. vastifica*									
EA276	KY655377	*Spongiobacternickelotolerans*OOP-Ni033-1-1-2(T)	98.8	++	++	-	-	-	-
^a^Identification based on partial 16S rRNA gene sequence analyses of all antagonistic bacteria.
^b^%similarity with closely related type strain.
^c^Antagonistic activity of all bacteria isolated in this study. The activity was measured after 3-5 days incubation at 28°C by measuring the clear zone of mycelial growth inhibition: -, Negative; +, 3 mm; ++, between 4 to 6mm; +++, between 7 to 9mm; ++++, between 10 to 12 +++++, between 13 to 15.
^d^Antibacterial activity against human pathogenic bacteria: -, Negative; +, 2-3 mm.
